# Non Inflammatory Boronate Based Glucose-Responsive Insulin Delivery Systems

**DOI:** 10.1371/journal.pone.0029585

**Published:** 2012-01-17

**Authors:** Indrani Dasgupta, Eric A. Tanifum, Mayank Srivastava, Sharangdhar S. Phatak, Claudio N. Cavasotto, Mostafa Analoui, Ananth Annapragada

**Affiliations:** 1 Department of Pediatric Radiology, Texas Children′s Hospital and Baylor College of Medicine, Houston, Texas, United States of America; 2 School of Biomedical Informatics, The University of Texas Health Sciences Center at Houston, Houston, Texas, United States of America; 3 Cense Biosciences Inc., Manvel, Texas, United States of America; Stanford University, United States of America

## Abstract

Boronic acids, known to bind diols, were screened to identify non-inflammatory cross-linkers for the preparation of glucose sensitive and insulin releasing agglomerates of liposomes (Agglomerated Vesicle Technology-AVT). This was done in order to select a suitable replacement for the previously used cross-linker, ConcanavalinA (ConA), a lectin known to have both toxic and inflammatory effects *in vivo*. Lead-compounds were selected from screens that involved testing for inflammatory potential, cytotoxicity and glucose-binding. These were then conjugated to insulin-encapsulating nanoparticles and agglomerated via sugar-boronate ester linkages to form AVTs. *In vitro*, the particles demonstrated triggered release of insulin upon exposure to physiologically relevant concentrations of glucose (10 mmoles/L–40 mmoles/L). The agglomerates were also shown to be responsive to multiple spikes in glucose levels over several hours, releasing insulin at a rate defined by the concentration of the glucose trigger.

## Introduction

The development of glucose-responsive controlled insulin delivery systems has attracted much attention due to its potential application in maintaining normal blood glucose levels, one of the key goals of the treatment of Type 1 diabetes [1]. Several self-regulated insulin delivery systems have been developed in recent years. These include polymers with sugar binding functional group (e.g. ConA, Phenylboronic acid) which form complexes with diols and polyols in aqueous environment [2], [3]. The critical solution behavior (e.g. LCST: lower critical solution temperature) of the polymers have been shown to change with environmental variations in glucose concentrations. Insulin is loaded with the polymers, and released, when the hydrophilicity of the complex is increased, due to the high-affinity binding of the glucose-responsive group to free glucose [3], [4]. Other types of delivery vehicles being explored are hydrogels containing chemically modified insulin, with glucose moieties binding to ConA or similar lectin, which can be dissociated when exposed to increasing concentrations of glucose thereby releasing the insulin [5], [6] and closed loop insulin delivery systems which contain a continuous glucose monitoring device that measures blood glucose concentration, a control algorithm to calculate the amount of insulin to be delivered, and an insulin pump. The route of insulin administration depends on whether the system is extracorporeal (subcutaneous monitoring and delivery) or implanted (intravenous monitoring and intraperitoneal delivery) [7].

Our group has previously advanced a platform technology for glucose responsive insulin delivery, the AVT or agglomerated vesicle technology. AVT is a chemically cross-linked agglomerate of liposomes loaded with insulin [8]. The cleavage of the chemical cross-links is tunable and can be triggered by the endogenous glucose concentration [9]. Thus, hyperglycemic events are quickly restored to euglycemic levels by cleaving of the cross-links followed by release of insulin encapsulated within the liposomes. Since the cleaving agent is endogenous glucose itself, the amount of insulin released is directly dependent on the blood glucose concentration and is halted soon after reaching normoglycemia, thereby avoiding the risk of hypoglycemia. Preliminary *in vivo* studies have been reported in previous studies from our group, establishing the drug release characteristics and stability of the AVTs [9], [10]. However, the major shortcoming of these AVT particles is that the glucose-sensitive linkage is based on competitive binding of ConA to a sugar. ConA is a lectin with high affinity for glucose but is known to be toxic [11]. To address this shortcoming we have carried out a screening study and identified a small molecule linker which binds to glucose with a binding constant similar to that of ConA but is less toxic and has lower inflammatory potential.

Boronic acids are known to bind diols, including glucose, fructose etc., reversibly, to form stable mono- and bisdentate complexes [12], [13], [14]. The saccharide binding property of boronic acids has prompted their use as sugar recognition moieties in glucose sensors [15], [16], [17] and self-regulated insulin delivery systems [18]. A sugar binding boronic acid can therefore be used to synthesize a boronic acid functionalized liposome which in turn can bind to glucose functionalized liposomes, thereby forming glucose-cleavable AVTs. Our goal therefore, was to identify compounds that could bind to a wide variety of sugars with a range of binding constants, both above and below that of glucose, so that the AVT particles are capable of cleaving over a broad range of glucose concentrations. Further, *in vivo* use of an AVT particle based on this compound would require that the compound (and the resulting particle) have low toxicity and inflammatory potential. In this study therefore, as a first step to this goal, we screened a large number of boronic acid derivatives for their toxicity and inflammatory properties and identified lead compounds that could safely be used *in vivo*. Nuclear translocation of NF-κB was used as an indicator of the inflammatory potential of the boronic acids. NF-κB is a widely studied transcription factor which plays a key role in inflammatory response [19]. The NF-κB family includes the following proteins: RelA(p65), RelB, RelC (cRel), NF-κB1 (p50 and p105) and NF-κB2 (p52 and p100) [20]. Under normal conditions the RelA-p50 heterodimers are present in the cytoplasm, bound to proteins of the IκB family. Upon stimulation by a wide range of stimuli such as tumor necrosis factor α (TNFα), interleukin 1 (IL1) or other pro-inflammatory species, the IκB proteins are phosphporylated by the IKK complex. This leads to release and subsequent nuclear translocation of the RelA-p50 dimers. The translocated RelA-p50 dimers activate the transcription of various inflammatory cytokine genes (eg. IL8, IL12 etc.). The nuclear translocation of NF-κB occurs within the first 30–60 mins of exposure to a pro-inflammatory species and is an early prognosticator of the downstream cascade of events. Therefore, measurement of nuclear translocation of NF-κB, upon exposure to a test species, provides an early and sensitive indication of its inflammatory potential.

**Figure 1:**
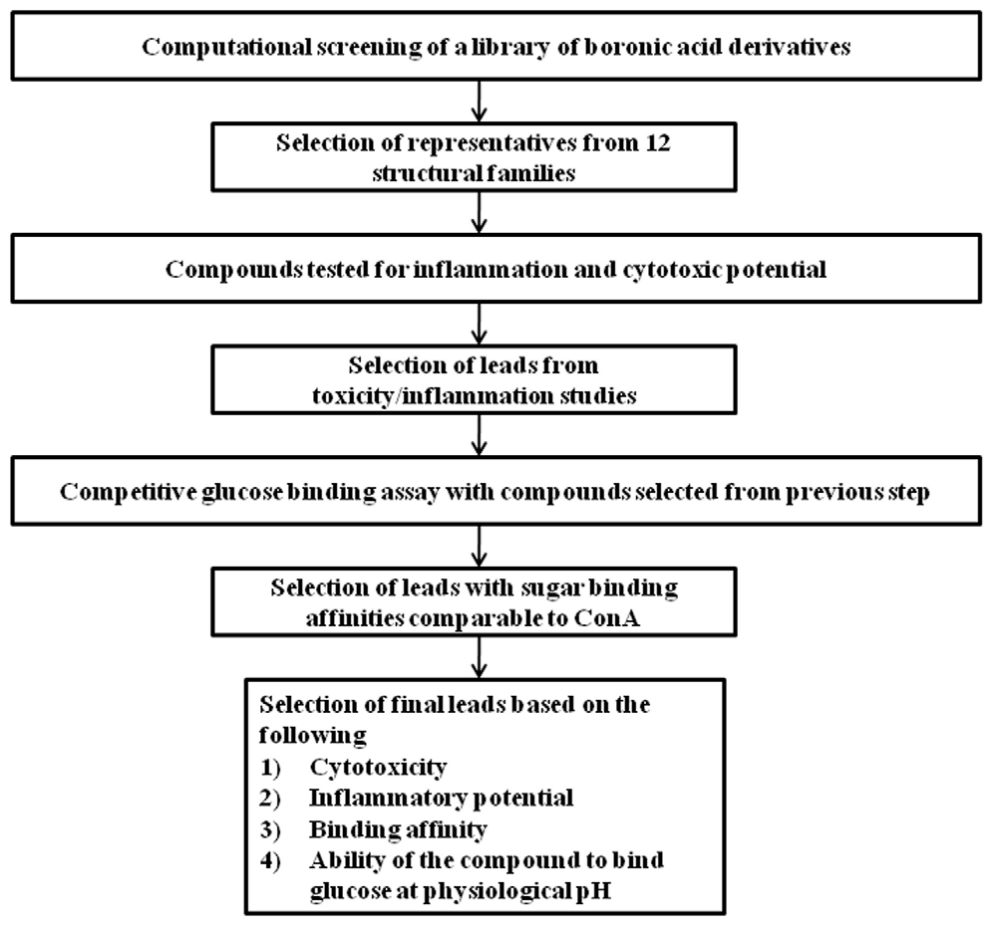
Flowchart describing the experimental screening and selection of boronic acid linker for insulin AVT.

The sugar binding affinities of the lead compounds were compared with those of ConA to identify a suitable replacement for use in the next generation of AVT particles. The steps involved in the screening process are summarized in Figure 1.

After completion of the screening, several boronic acids, mainly belonging to two structural families, were identified as lead candidates for replacing ConA. The selected candidates were incorporated on the surfaces of liposomes, and used as a replacement of ConA for synthesizing insulin loaded AVTs. The *in vitro* insulin release profiles of the boronic acid-AVTs were also tested and compared with that of the ConA-AVT.

## Results

### Selection of Boronic acids for screening

The first step in the screening process was a computational clustering to select bi-functional boronic acid compounds with reactive side chains for conjugation with lipid molecules. This was necessary to identify boronic acids which can be used to functionalize the insulin loaded liposomes and covalently link them with other liposomes which have sugar molecules on their surface.

A boronic acid library of 469 compounds was clustered into 150 groups using the number of rings and the number of aromatic rings as criteria and applying the Extended Connectivity Fingerprint algorithm with 4 bond layers (ECFP4) within Pipeline Pilot [21], [22]. The ECFPs account for the environment of every atom in a molecule in an iterative way until a given threshold, thus being able to essentially represent an infinite number of structural features. For the numeric properties we chose “Number of Rings” and “Number of Aromatic Rings”, since one of the goals was to cluster the compounds based on the number of ring structures. The cluster center selection parameter was set to Maximum Dissimilarity and the Euclidean (RMS Distance) option was set as the choice for the Numeric Distance Function parameter. The Number of Clusters parameter was set to 150. The 150 clusters, obtained using the process described above, were further reclassified by visual inspection into two datasets of 76 (333 compounds) and 74 (136 compounds) clusters. This reclassification was done in order to separate singletons or clusters containing fused ring compounds. Only the first set was selected for further development. From each cluster, one or more compounds were visually selected, so as to maximize the diversity in terms of ring type (benzyl or pyridyl), and number, position (ortho, meta, para) and type of substituents. The final library of 110 test compounds was then purchased from Sigma-Aldrich, MO.

The compounds in the library were further grouped as derivatives of the following structural families:1)Phenylboronic acid 2) Pyridine 3) Napthalene 4) Indole 5) Thiophene 6) Thianthrene 7) Cyclopropyl 8) Pyrrole 9) Isoquinoline 10) Oxazole 11) Pyrazole and 12) Dibenzofuran.

### Toxicity and inflammatory potential

Little is known about cytotoxic and inflammatory properties of boronic acids. In this study we evaluated the inflammatory potential and toxicity of the boronic acids selected from the computational screening, using NF-κB translocation (PCC values) and MTT assays (Cell surviving fraction) respectively. It was found that majority of the compounds do not cause nuclear translocation of NF-κB or substantial loss of cell survival, when exposed at the lowest of the three doses (40 nM) that were tested. Any compound that showed a positive PCC value at 40 nM and/or lower than 85% cell survival at the same were eliminated.

It was also observed that when used at the highest concentration (160 nM), 50 out of the 70 compounds returned a PCC value over 0.1, indicating inflammation and 38 of them led to cell survival fraction <50%.

**Figure 2:**
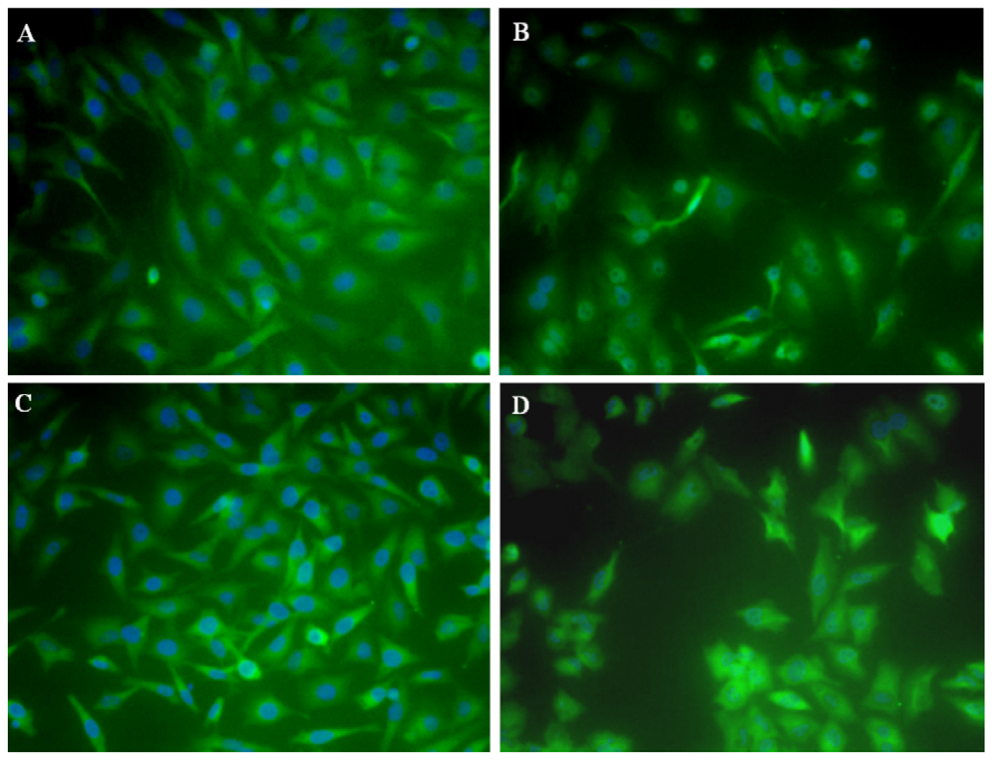
Effect of boronic acid treatment on the nuclear or cytoplasmic localization of NF-κB. Nuclear stain DAPI is shown in blue, while the NF-κB is represented by green. Visible blue nuclei surrounded by green indicate cytoplasmic NF-κB, while obliteration of the blue by green signal in the nucleus indicates nuclear NF-κB. (A) Untreated control, cytoplasmic NF-κB (B) Positive control, nuclear translocation of NF-κB in cells treated with LPS, (C) Treatment with 2,4-ditertbutoxypyrimidine-5-yl-boronic acid (80 nM, 2 hrs.), predominantly cytplasmic NF-κB and (D) Treatment with 5-isoquinoline-boronic acid (80 nM, 2 hrs.), predominantly nuclear NF-κB.

Hence, stress was given on the PCC values and surviving fraction obtained when cells were treated with the intermediate dose of 80 nM. (Representative images from the NF-κB nuclear translocation assay are shown in Figure 2.) The criteria for selection of compound were the following:

1. A PCC value ≤0.1 at 80 nM
2. Cell Surviving Fraction ≥80% at 80 nM.

The compounds listed below, belonging primarily to two different structural families, were found to fulfill the selection criteria outlined above and were chosen for the next step which is binding affinity determination:

- Phenylboronic acid derivatives
  2,4-dichlorophenylboronic acid
  4-aminocarbonylphenylboronic acid
  3-N,N-dimethylphenylboronic acid
  4-hydroxyphenylboronic acid
  4-propylphenylboronic acid
  4-chlorocarbonylphenylboronic acid
  3-[(E)-2-nitrovinylphenylboronic acid
- Pyridine and Pyrimidine boronic acid derivatives
  2-bromopyridine-3-boronic acid
  2,4-ditert-butoxypyrimidin-5-yl-boronic acid
  2,4-bis(benzyloxy)pyrimidin-5-boronic acid

Another boronate from this family, 2-bromopyridine-5-boronic acid, also passed the selection criteria mentioned above. However when compared with the closely related derivative, 2-bromopyridine-3-boronic acid, the latter was found to have a much better cell survival at 160 nM (88%) and was chosen instead.

In addition, three compounds that did not belong to either of these families were selected. These were:

cyclopenten-1-yl-boronic acid
5-phenyl-2-thienylboronic acid
5-formylthiophene-3-boronic acid.

### Binding Assays

The binding affinity of the boronic acids to glucose was determined by a competition assay using ConA, a known high affinity glucose binder. The assay was initiated by immobilizing glucose molecules on the surface of activated magnetic beads, as described in the methods.The binding reaction was carried out in a 96 well plate by incubating the beads containing the glucose molecules on their surface, with a fixed concentration of ConA-FITC (2.4 µM) and increasing concentrations of the boronic acid being tested (2.5 µM–20 µM). After completion of the reaction, the beads were washed to remove excess unbound ConA-FITC and boronic acid. The fluorescence intensity of the mixture was measured and used as an indication of the amount of ConA-FITC bound to the glucose molecules on the surface of the beads, or [ConAs]. The ConA-FITC-glucose interaction is expected to be inhibited by boronic acid which competes and binds to the glucose molecules and displaces the bound ConA-FITC, thereby causing a decrease in fluorescence intensity of the mixture. The reduction of fluorescence intensity can be co-related to [ConAs] by Equation 1, where F_max_ is the maximal fluorescence intensity (positive control; ConA-FITC only), F_obs_ is the observed fluorescence intensity for the test wells.
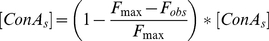
(Eq. 1)


The relationship between the amount of adsorbed ConA ([ConAs]) and the amount of boronic acids [BA] is given by Equation2, where S_t_ is the concentration of glucose binding sites, Ka*_ConA_* is the equilibrium constant for the binding of ConA-FITC and glucose and Ka*_BA_* is the equilibrium constant for binding of Boronic acid and glucose.
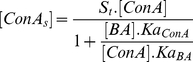
(Eq. 2)


Simple rearrangement yields Equation 3, from which the ratio of the two equilibrium constants (Ka*_ConA_*/Ka*_BA_*) can be derived
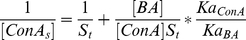
(Eq. 3)


**Figure 3:**
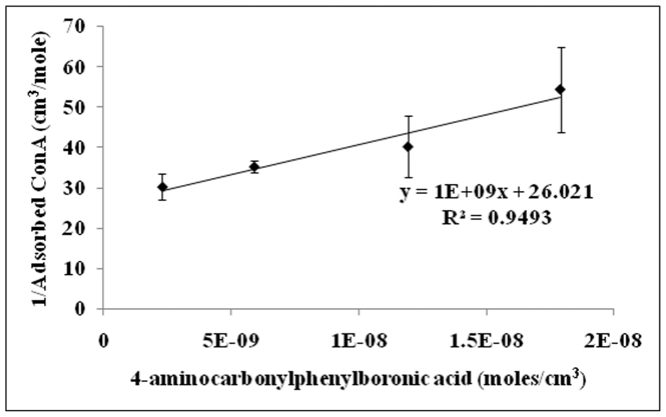
Determination of binding affinity. [ConAs] = concentration of ConA-FITC adsorbed to sugar molecules on the surface of magnetic beads., [ConA] = bulk concentration of ConA-FITC, [St] = Total sugar sites available for binding of ConA-FITC, Ka_ConA_ = Binding constant of ConA and glucose, Ka_BA_ = Binding constant of boronic acid and glucose; The ratio of Ka_ConA_ and Ka_BA_ can be calculated multiplying [St], [ConA] and the slope of the plot.

The concentration of sugar sites (S_t_) can be calculated from the intercept of a plot of 1/[ConAs] vs. 1/[BA]. Figure 3 shows a representative plot for the binding constant calculations. The competition assay was carried out for all the thirteen compounds selected from the toxicity and inflammation screen. Three of those compounds did not show any competitive displacement of ConA-FITC and were thus removed from the screen. Using the methods described above, we could determine the binding constants for the remaining ten boronic acids.

### Selection of final leads

**Figure 4:**
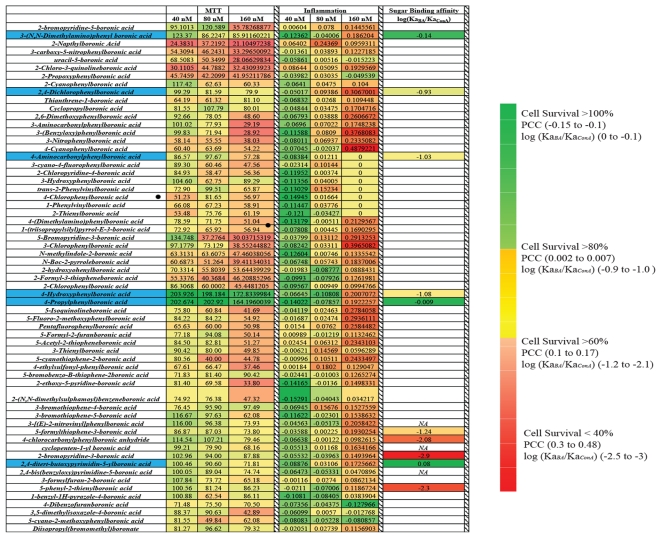
Heat map showing the Cytotoxicity (MTT), Inflammation (PCC) for all the boronic acids. *Relative Sugar binding affinities*, compared to ConA, are shown for compounds selected from the first two screening steps.The final leadsselected compounds are highlighted in blue.

The results for the overall screening of the boronic acids chemical library are summarized as a color intensity map in Figure 4. The final leads, highlighted in blue, have the following properties

1. Cell Surviving fraction
  - 85% at 40 nM
  - 80% at 80 nM
  - 55% at 160 nM
2. Inflammatory potential, as indicated by PCC values.
  - <0 at 40 nM
  - <0.1 at 80 nM
  - ≤0.3 at 160 nM
3. Sugar binding affinity relative to ConA, quantified by the log of the ratio between the glucose binding constants derived for the Boronic Acids (Ka*_BA_*) and ConA (Ka*_ConA_*).
  - Ranged between −1.08 and 0.08.

These compounds can be broadly grouped into two structural families: derivatives of phenylboronic acid with electron withdrawing substituents and pyrimidineboronic acid. The binding constants (Ka) for the six lead boronic acids are shown in Table 1, along with the surviving cell fraction and correlation between nuclear and cytoplasmic fractions of NF-κB when HeLa cells were treated with these compounds.

**Table 1:** Lead Boronic Acids.

Boronic Acid	Glucose Binding Affinity (M^−1^)	Cell Survival (80 nM) (%)	PCC(80 nM)
4-aminocarbonylphenyl boronic acid	45	97	0.01
3-N,N-dimethylamino phenyl boronic acid	357	86	−0.04
4-propylphenylboronic acid	303	202	−0.07
2,4-dichlorophenyl boronic acid	58	81	0.09
4-hydroxyphenyl boronic acid	80	198	−0.01
2,4-ditertbutoxypyrimidin-5-yl-boronic acid	608	90	0.03

The binding constants (M^−1^), HeLa Cell Survival (%)** and PCC** values for the lead boronic acids (** Values shown are for HeLa cells incubated for 2 hrs. at 37°C with 80 nM concentration of the compounds).

### Preparation of lipid-PEG-boronic acid and lipid-PEG-sugar conjugates

To prepare liposomes functionalized with the lead compounds, PEGylated lipid conjugates were synthesized and incorporated in the liposomal formulation. The conjugation of the boronic acids with lipid-PEG molecule is not expected to interfere with their ability to bind glucose for the following reasons: (a) attachment to the PEG moiety (which floats freely on the surface of the liposome in the final formulation), is away from the B(OH)_2_ group involved in complexation with the diols [14]; and (b) PEGylated boronic acids have been shown to effectively bind glucose in hydrogel and polymer based glucose sensing devices [23], [24]. It should also be noted that the boronic acids were not extensively PEGylated, rather only a single lipid-PEG molecule was used per boronic acid.

**Figure 5:**
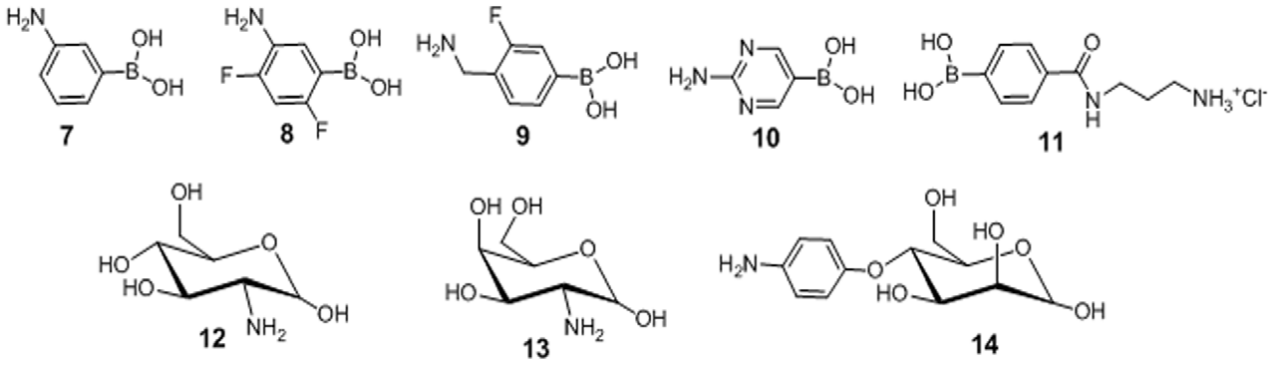
Amine substrates employed in PEGylated lipid-boronics acid and sugars conjugate preparation.

Five amine functionalized boronic acids (Figure 5,compounds 7–11), with similar electronic properties as the lead compounds, were selected for facile conjugation to the carboxyPEGylated lipid. Each was allowed to react with DSPE-PEG-COOH under carbodiimde conditions as shown by the example in Scheme S1. In a similar manner, amine derivaties of glucose (12), galactose (13) and mannose (14) were also conjugated to DSPE-PEG-COOH under similar conditions (Scheme S2), and used in the formulation of sugar coated liposomes.

**Figure 6:**
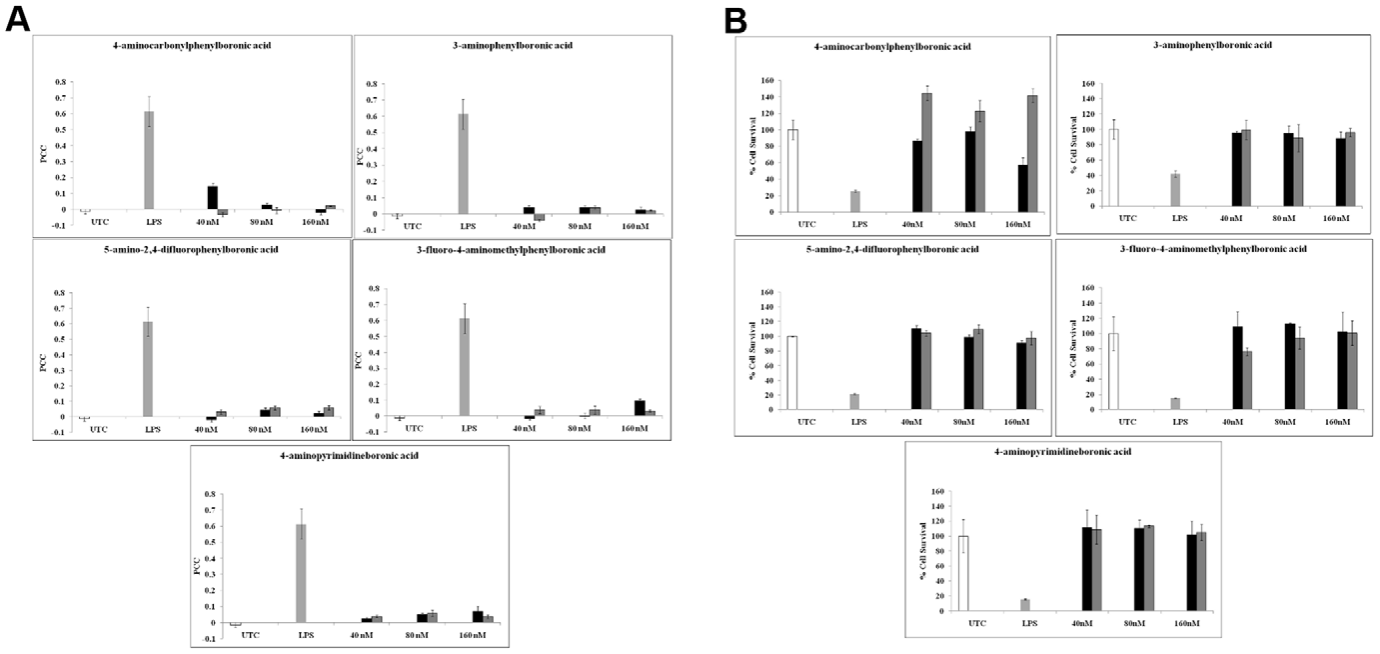
Effect of PEGylation on cytotoxicity and inflammatory potential of boronic acids. (A) Pearson's Correlation Coefficient between the nuclear and cytoplasmic fractions of NF-κB molecule in HeLa cells treated with the lead boronic acids [both as single molecule (black bars) and conjugated to DSPE-PEG-COOH(dark gray bars)], (B) Surviving fractions of HeLa cells treated with the same compounds as in (a). UTC: Untreated Control, LPS: Lipopolysachharide.

The inflammatory potential and toxicity of these newly synthesized lipid-PEG-boronate conjugates were also assayed and the results are shown in Figure 6A and 6B. One of the compounds, 3-fluoro-4-aminomethylphenylboronic acid caused enhanced toxicity whereas 4-aminocarbonylphenylboronic acid, showed higher cell survival rates and lower inflammatory potential than it's non-conjugated form.

### Synthesis of Boronic Acid-AVTs and *in vitro* release of Insulin

#### Synthesis of boronic acid-AVTs

Two different types of liposomes (sugar linked and boronic acid linked) were prepared as described in the methods section. A 40 mol% concentration of cholesterol was added to the lipid bilayer. The cholesterol is known to broaden the pretransition and main phase transition of DPPC bilayers [25], [26]. This ensures that the membrane is only softened at physiological temperature (∼37°C) and does not undergo a sharp melting transition. These were co-incubated to form agglomerates. The optimal conditions for agglomeration (molar excess of the boronic acid liposome relative to the sugar liposomes, pH and incubation time) were different for each one of the boronate-AVTs as shown in Table 2. The results indicate that these boronic acids are capable of forming the cross-links under near physiological pH.

**Table 2:** Optimal conditions for preparing boronic acid AVTs.

Boronic Acid Moiety	Sugar Liposome: Boronic Acid Liposome (mole/mole)	pH
3-aminophenylboronic acid	1∶5	7.5
5-amino-2,4-difluorophenylboronic acid	1∶5	8
4-aminocarbonylphenylnboronic acid	1∶20	8
3-fluoro-4-aminomethylphenylboronic acid	1∶10	8

To confirm that the AVTs were formed by chemical cross-linking of the boronic acid and sugar moieties, the agglomerates were exposed to 10 mM glucose. The agglomerates were readily cleaved in the presence of glucose, due to competitive binding of the boronic acids with the free glucose. This was demonstrated by the increase in fraction of particles sized under 1 µm from 13% to 37% upon incubation with glucose.

#### *In vitro* release of Insulin from the boronic acid-AVTs

**Figure 7:**
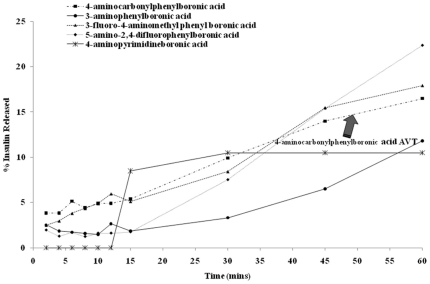
Cumulative release of insulin from boronate-AVTs in the absence of glucose trigger.

A small volume (500 µL) of the boronate-AVT was loaded inside a tubular dialysis membrane (100 K MWCO), sealed with clips, and dialyzed against a PBS solution (pH 7.4) for 60 mins to monitor the passive release of insulin without trigger. Aliquots were removed from the external phase at regular intervals and insulin concentration was assayed by reading the absorbance at 214 nm. Figure 7 shows the cumulative release of insulin from all of the boronate-AVTs listed in Table 2. The AVTs synthesized with the boronic acids containing strong electron withdrawing groups (3-fluoro-4-aminomethylphenylboronic acid and 5-amino-2,4-difluorophenylboronic acid) were found to release their insulin content too rapidly. The 4-aminopyrimidineboronic acid AVT showed one episode of rapid burst release and did not release any insulin after that. The two lead candidates selected from this assay were (a) 3-aminophenylboronic acid AVT and (b) 4-aminocarbonylphenylboronic acid AVT releasing 12% and 16% of their insulin content respectively. Out of these two, the 4-aminocarbonylphenylboronic acid AVT was chosen as the final lead based on our previous observation that the toxicity and inflammatory potential of the parent compound is reduced upon conjugation with PEGylated lipid (Figure 6A and 6B).

**Figure 8:**
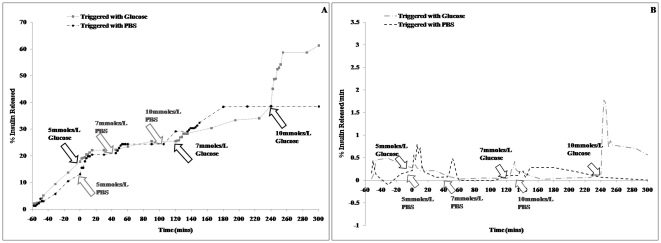
In-vitro release of Insulin from Boronic Acid AVT. (A) Cumulative and (B) differential plots for release of insulin from 4-aminocarbonylphenylboronic acid AVTs triggered with 5 mM, 7 mM and 10 mM glucose.

Insulin release from the lead AVT, upon addition of varying concentrations of glucose trigger, was then assayed for several hours. The trigger concentrations were chosen so as to mimic blood glucose levels at normoglycemic and hyperglycemic conditions. A control assay was also set up where the lead AVT was triggered with PBS instead of glucose. The agglomerate exhibits a burst release of insulin within 2 mins after introduction of the glucose trigger (Figure 8A and 8B). The release rate slows down over time until another dose of glucose is added, which triggers a new episode of burst release. The minimum concentration of glucose required for cleaving the agglomerates and releasing insulin is 10 mmoles/L, which corresponds to a blood glucose level of 180 mg/dL. We did not observe a burst release of insulin when the particles were triggered with glucose concentrations analogous to hypoglycemia (5 mmoles/L, ∼90 mg/dL) and normoglycemia (7 mmoles/L, ∼126 mg/dL), suggesting that the AVTs are suitable for maintaining normal blood glucose levels (Figure 8B). Also, the insulin release rate from these microparticles is dependent on the glucose concentration, thereby making this platform potentially useful in avoiding hyperinsulinemia and corresponding hypoglycemia.

## Discussion

In this work, we have screened a library of boronic acid derivatives for toxic and inflammatory properties and identified a lead candidate molecule and several other potential backups that will be better suited for *in vivo* usage as an alternative to the lectin,ConA,in a glucose-responsive, insulin releasing composite microparticle delivery system, AVT [8], [9]. The glucose binding affinities of the boronic acids short-listed from the toxicity and inflammation screen were also determined; to ensure selection of a compound that is similar to ConA in terms of sensitivity towards variations in glucose levels.The selected leads were re-evaluated for inflammation and cytotoxicity after being conjugated with PEGylated lipids, for incorporation into the liposomes. This was done to ascertain that the inflammation and toxicity profile of the single molecules were not perturbed by the lipid-PEG conjugation. Surprisingly we found that some of the lipid-PEG conjugated boronic acids were even less toxic and inflammatory compared to their monomeric forms as shown in Figure 6.This is a critical observation as chronic exposure to the AVTs from daily subcutaneous injections may result in long term effects, and the reduction in toxicity by lipid-PEG conjugation offers a promising solution to this problem.

**Figure 9:**
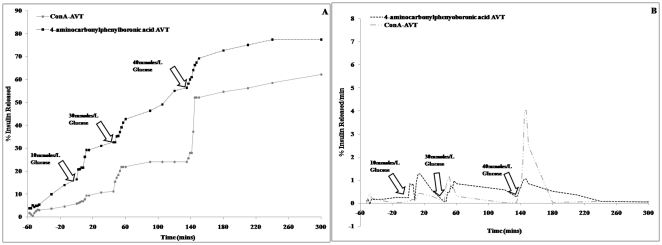
In-vitro release of Insulin from Boronic Acid and ConA AVTs. Cumulative and differential plots for insulin release from 4-aminocarbonylphenylboronic acid AVT and ConA AVTs triggered with 10 mM, 30 mM and 40 mM glucose.

To establish that these particles can be used for building a insulin delivery vehicle we have performed preliminary *in vitro* release studies. Parent liposomes were fabricated to contain either sugar (glucose, galactose and mannopyranside) or one of the lead boronic acid molecules on the outer surface. The liposomal formulations were mixed together at room temperature to induce reversible ester formation between the boronic acid and the 1,2-cis-diol moieties of the sugar molecules. This resulted in cross-linking of the liposomes to form agglomerated vesicles (AVT). We have shown that *in vitro* cleavage of the AVTs by 10 mmoles/L glucose trigger can cause a burst release of insulin. This is followed by a slow release over several hours without further glucose stimulation. This basal release rate is a critically important property of these particles. In the absence of a basal untriggered release, the only release of drug will be in the event of a trigger. The amount of drug residual in the delivered dose on any given day will then be dependent on the trigger history, and leaves open the potential for significant carryover from day to day. This could lead to dose-buildup, and catastrophic consequences. Thus, a basal release rate, that will insure dose depletion in a given day, regardless of triggering, is a critical requirement for these particles. Indeed, a shortcoming of the ConA based particles shown in Figure 9 is the absence of such a basal release rate.

The release rate however can be increased very quickly by a spike in glucose concentration (Figure 8A and 8B). In vivo, this would translate to the ability of the particles to control blood glucose levels for long periods, in spite of periodic glucose excursions, thus reducing the need for multiple daily injections. We have also demonstrated that the system is capable of responding to severely hyperglycemic [27] glucose levels of 30 mmoles/L and above (Figure 9A and 9B). The pattern of insulin release is similar to that of the ConA AVTs (Figure 9). Further studies will be conducted to optimize the insulin release from these boronic acid linked liposomes both *in vitro* and *in vivo*. Current results indicate that phenylboronic acids with electron withdrawing substituents at the 2- and 4- positions of the phenyl ring form very leaky AVTs and are not suitable for triggered release applications (Figure 7). However an interesting future study can be the use of the multiple lead candidates identified from the screening efforts to prepare liposomes with more than one boronic acid on their surface, (See Table 1), differing in their affinity towards glucose. This will allow us to prepare AVTs which are held together by boronic acid-diol linkages of varying strengths. The degree of AVT cleavage and the rate of insulin release will then depend on the number of strong, weak and moderate linkages present. This way the AVTs can be fine-tuned to be sensitive to even minor fluctuations in glucose levels.

## Materials and Methods

### 1) NF-κB assay for inflammation

The inflammatory potential of the compounds was studied by measuring the nuclear translocation of NF-κB in HeLa cells by immunocytochemistry and high throughput high content microscopy. 15000 HeLa cells were plated in 96 well plates the night before the experiments. On the day of experiment the cells were treated with the test compounds at three different concentrations for 2 hrs. At the end of the incubation cells were washed with PBS and were fixed in 4% paraformaldehyde for 15 mins and subsequently permeabilized with 0.01% Triton X-100 in PBS for 10 mins. The cells were washed with PBS 3 times. Nonspecific sites were blocked with 5% BSA in PBS and then incubated with anti-NF-κB (Rabbit polyclonal NF-κB p65(C-20), Santa Cruz Biotechnology, ®Inc.,Santa Cruz, CA) for 1 hr followed by incubation with FITC-labeled secondary antibody (Goat α-rabbit IgG-FITC labeled, Santa Cruz Biotechnology, ®Inc., Santa Cruz, CA). After washing the cells at the end of the incubation they were treated with DAPI for 1 min and kept at 4°C until further analysis. Images of the cells were acquired with a Beckman-Coulter IC 100 automated high-throughput microscope system and analyzed using Cyteseer software (Vala Sciences, San Diego, CA) [28], [29]. The co-localization of NF-κB was quantified by measuring the Pearson's correlation coefficient (PCC) between the nuclear and cytoplasmic fraction of the NF-κB molecule. The PCC is a measure of the overlap between the pixel intensities of the nuclear and cytoplasmic NF-κB images of the same cell, and is calculated as [30]:
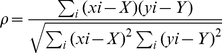
(Eq.4)


Where *xi* and *yi* are the individual pixel intensities corresponding to the nuclear and cytoplasmic NF-κBfluorescence, respectively; *X, Y* are the corresponding mean intensities. The PCC values can range from −1 to 1. A positive correlation (positive PCC value) indicates nuclear translocation of NF-κB. A negative correlation (negative PCC value) indicates an absence of nuclear translocation [30].

### 2) MTT assay for cytotoxicity

The cytotoxicity of the compounds was studied by MTT assay. 15000 HeLa cells were plated in 96 well plates on the night before the experiment. On the day of experiment the cells were treated with the test compounds at three different concentrations for 2 hrs. At the end of the incubation MTT assays (*In vitro* toxicology MTT based assay kit, Sigma-Aldrich, MO) were performed according to the manufacturer's protocol.

### 3) Binding assay

The binding affinity of the boronic acids to glucose was determined by a three component –competitive assay which includes a fluorescently labeled ConA, Boronic Acids and Glucose. Glucose molecules were immobilized on a solid support using the following method: Carboxy terminated magnetic beads(Bangs Laboratories, Inc., Fisher, IN) were activated by suspending them in 100 mM MES buffer (pH 4.5) and aliquoted (100 µl/well) in 96 well plates. The activated beads were conjugated to amine terminated glucose molecules (100 mg/ml), D-(+)- glucosamine (Sigma-Aldrich, MO) [31] using EDC as a cross linker. The cross-linking reaction was run for 24 hr, at room temperature on an orbital shaker, and after completion the beads were separated by placing the microplate on a magnetic separator and washed thoroughly with PBS at pH 7.2 to remove unbound glucose, excess EDC and Sulfo-NHS.

The competitive binding, between ConA and Boronic acid, was then carried out as follows:

The magnetic beads, decorated with glucose molecules on their surface as described above,were then co-incubated for 1 hour with varying concentrations of the boronic acid (2.5 µM–20 µM) being tested and a fixed concentration of fluorescently tagged ConA, 2.4 µM (ConA-FITC, Sigma Aldrich, MO). Non-conjugated magnetic beads were treated with ConA-FITC to determine non-specific binding. Maximal binding was determined by treating glucose conjugated beads with ConA-FITC only.

The beads were washed 3 times with PBS at pH 7.2 to remove unbound boronic acids and ConA-FITC and resuspended in PBS and stirred for 15 mins at room temperature before measuring FITC fluorescence (excitation: 495 nm, top emission: 520 nm; 6 flashes per well; average of n = 3 wells) in a Flexstation II^384^ microplate reader (Molecular Devices, Inc., Sunnyvale, CA) to quantify the amount of glucose bound to the boronic acids.

### 4) Synthesis of boronic acid and sugar conjugates

All lipid-PEG-linker-boronic acid conjugates were prepared by coupling the amine-derivative of the boronic acid-linker (Figure 5), with DSPE-PEG-COOH [1,2-distearoyl-sn-glycero-3-phosphoethanolamine-N-(carboxy(polyethylene glycol)-2000), Avanti Polar Lipids, Inc., Alabaster, AL] using carbodiimide chemistry. 3-aminophenylboronic acid (7) was obtained from Sigma-Aldrich, St. Louis, MO; 3-fluoro-4-aminomethylphenylboronic acid (9), 5-amino-2,4-difluorophenylboronic acid (8) and 4-aminopyrimidineboronic acid (10) boronic acids were obtained from Combi-Blocks, Inc., San Diego, CA;. 3-aminopropyl tethered 4-aminocarbonylphenylboronic acid (11) was prepared in-house from 4-bromobenzoyl chloride and *N*-Boc-1,3-propanediamine in three steps. The NMR spectra are shown in Figues S1, S2 and S3.

In general, 50 mg of DSPE-PEG-COOH were dissolved in 2 ml anhydrous DMF (Sigma Aldrich, St. Louis, MO) followed by the addition of EDC (Pierce, Rockford, IL) (2.0 equivalents) and NHS (Pierce, Rockforfd, IL) (3.0 equivalents), and the mixture stirred for 30 mins at room temperature. The amine derivative of the boronic acid (2.0 equivalents) was then added and the reaction mixture stirred overnight. Amines obtained in the form of the ammonium salt, were desalted by stirring in 500 µL DMF and 35 µL triethylamine (Sigma Aldrich, St. Louis, MO) for 30 mins at room temperature prior to addition to the reaction mixture. The reaction mixture was then diluted with 4 mL MES buffer (50 mM, pH 4.18), transferred into a 2 K MWCO dialysis cassette (Pierce, Rockford, IL) and dialyzed twice against 2 L of the same buffer, then twice against 2 L water followed by freeze drying to obtain the desired conjugates.

**Figure 10:**
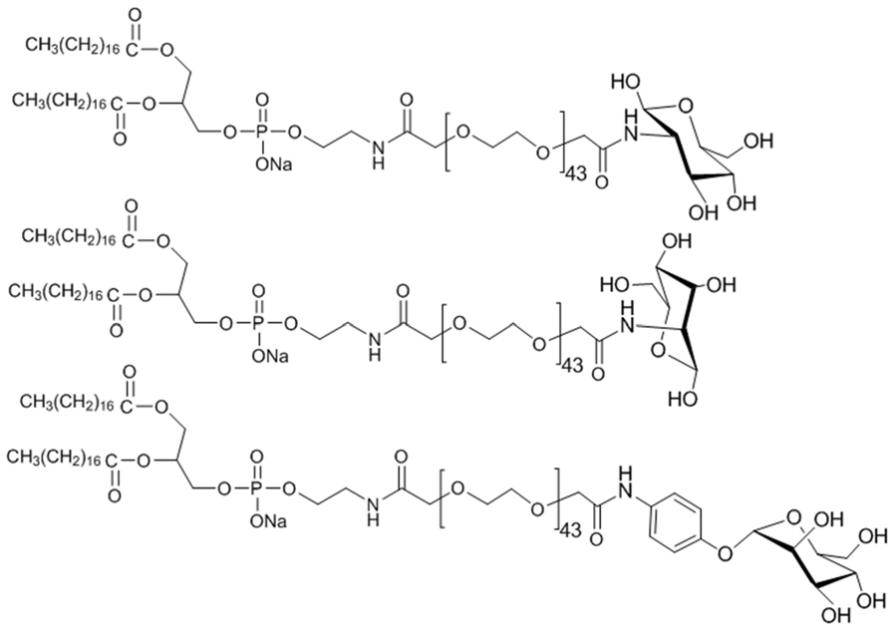
The final structure of DSPE-PEG-Sugar conjugates.

The lipid-PEG-sugar species were synthesized by conjugating the carboxyl group of DSPE-PEG-COOH to the amino group of glucosamine (12), galactosamine (13), and mannopyranoside (14) (Fisher Scientific, Houston, TX) (Figure 10), using the same protocol.

### 5) Synthesis of Liposomes functionalized with sugar/boronic acids and their agglomeration:

#### AVT particles with Sugar-ConA linkages were prepared using the following method

Human recombinant insulin (Sigma Aldrich, St. Louis, MO) was dissolved in Citrate Buffer (100 mM, pH 2.5) to a concentration of 15 mg/ml. The phospholipids DPPC (1,2-diplamitoyl-sn-glycero-3-phosphatidylcholine) and Cholesterol were purchased from Lipoid GMBH, Ludwigshafen, Germany. Lipids (56.4 mole% DPPC, 40 mole% Cholesterol and 1.2 mole% each of DSPE-PEG-Glucose, DSPE-PEG-Galactose and DSPE-PEG-Mannopyranoside) were dissolved in ethanol and hydrated with the insulin solution at 50°C for 15 mins. The final lipid concentration was 50 mM. The hydrated mixture was then passed 8 times through a 400 nm Nucleopore track-etch membrane at 50°C and a pressure of 100 psi.The parent liposomes had a mean diameter of 244.1±0.1 nm and a lipid concentration of 50 mM. Insulin was encapsulated inside the liposomes by passive loading. The pH of the liposomal formulation was maintained at 5.6 (isoelectric point of insulin). The liposomes were dialyzed against citrate buffer (100 mM, pH 5.6) to remove the unencapsulated insulin.The pH of the formulation was then raised to 6.6 and 3 mM CaCl_2_/3 mM MnCl_2_ were added for optimal binding of ConA. An aliquot of ConA solution (pH 6.6, with 3 mM CaCl_2_, 3 mM MnCl_2_) was added to the liposomal formulation at a 2∶1 molar ratio over the glycosyl species present in the outer leaflet of the lipid bilayer. The agglomeration process was completed in 10 mins.

#### AVT particles with boronic acid linkages were prepared using the following method

Lipid-PEG-boronic acid was synthesized by conjugating the carboxyl group of DSPE-PEG-COOH to the amine functionalized boronic acids using the carbodiimide coupling chemistry as described earlier. The lipid composition for the boronic acid functionalized liposome was the following: (56.4 mole % DPPC, 40 mole% Cholesterol and 3.6 mole% of DSPE-PEG-boronic acid). The insulin loading, extrusion and purification processes were similar to that used for the sugar liposomes as described above. The liposomes had a mean diameter of 184±0.162 nm.

#### Preparation of AVT

Two kinds of insulin loaded liposomal formulations, functionalized either with sugar molecules or a boronic acid derivative, were mixed and stirred at room temperature to form agglomerates. The mixtures were prepared using several molar ratios of sugar species to boronic acid species, ranging from 1∶2 to 1∶50, to determine the optimal excess of boronic acids required for agglomerate formation. The pH of the mixture was also varied between 7 and 11, the range in which boronic acids are known to bind diols [14], in order to select AVTs that are preferentially functional at physiological pH.

### 6) *In vitro* release of Insulin from the Boronate- AVTs

A small volume (500 µL) of the agglomerates was loaded inside a 100 K MWCO dialysis membrane (Spectrum Laboratories, Inc., Rancho Dominguez, CA) and dialyzed against PBS at pH 7.4 for 60 mins prior to cleaving with glucose to monitor the passive diffusion of insulin without trigger. This was followed by addition of glucose solution to the agglomerates inside the membrane at regular intervals to cleave the AVTs and trigger the release of insulin. Aliquots were removed from the external phase every 15 mins and insulin concentration was assayed by reading the absorbance at 214 nm.

The assay was continued for several hours to verify a halt in release of encapsulated insulin.

## Supporting Information

Figure S1 **4-aminocarbonylphenylboronic acid tethered to Boc-n-propylamine linker.**
(TIF)

Figure S2 **4-aminocarbonylphenylboronic acid tethered to n-propylamine linker.**
(TIF)

Figure S3 **4-aminocarbonylphenylboronic acid tethered to DSPE-PEG-COOH.**
(TIF)

Scheme S1 **General scheme for the preparation of PEGylated lipid-boronic acid conjugates.**
(DOC)

Scheme S2 **General scheme for the preparation of PEGylated lipid-sugar conjugates.**
(DOC)
