# Supplementary figures and images for: Non Inflammatory Boronate Based Glucose-Responsive Insulin Delivery Systems
Source: PLoS One. 2012 Jan 17;7(1):e29585. doi: 10.1371/journal.pone.0029585 (PMC3260138; doi:10.1371/journal.pone.0029585)

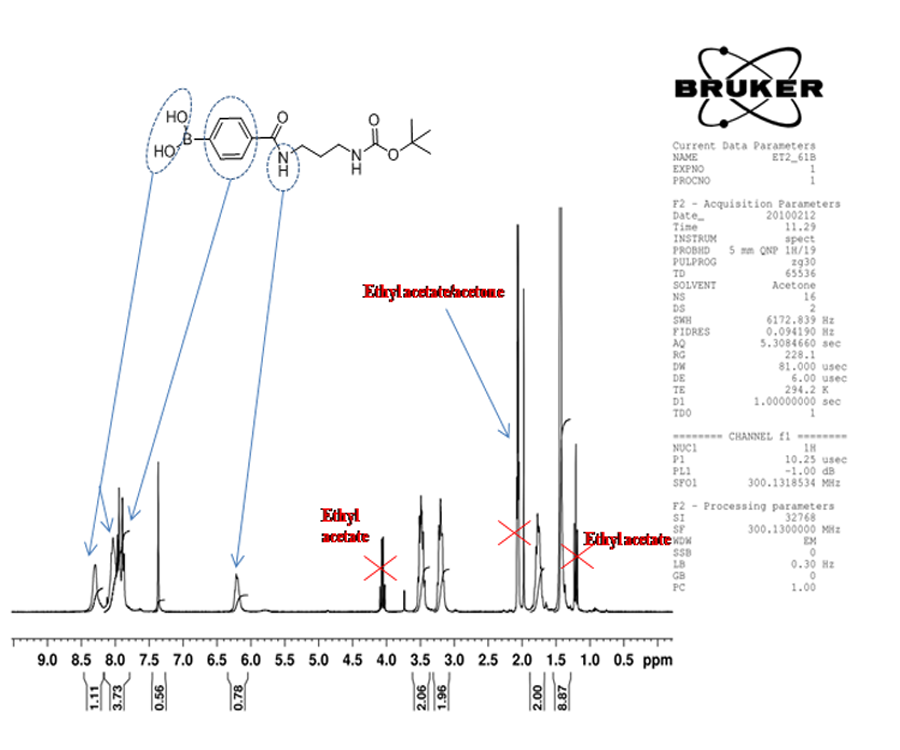

Supplement: Figure S1 — 4-aminocarbonylphenylboronic acid tethered to Boc-n-propylamine linker. (TIF) [file pone.0029585.s001.tif]

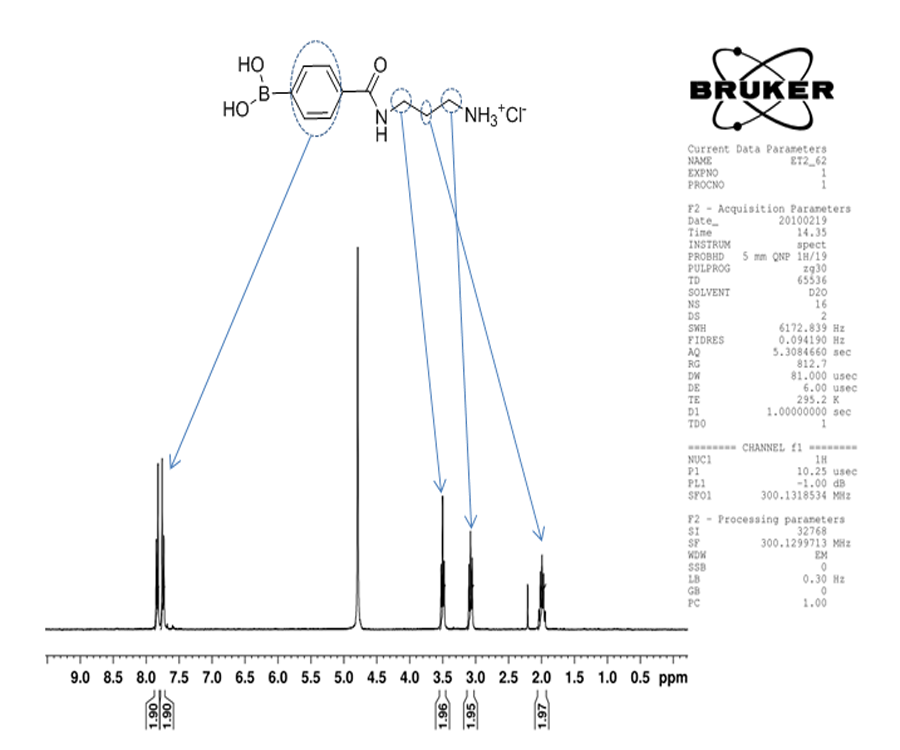

Supplement: Figure S2 — 4-aminocarbonylphenylboronic acid tethered to n-propylamine linker. (TIF) [file pone.0029585.s002.tif]

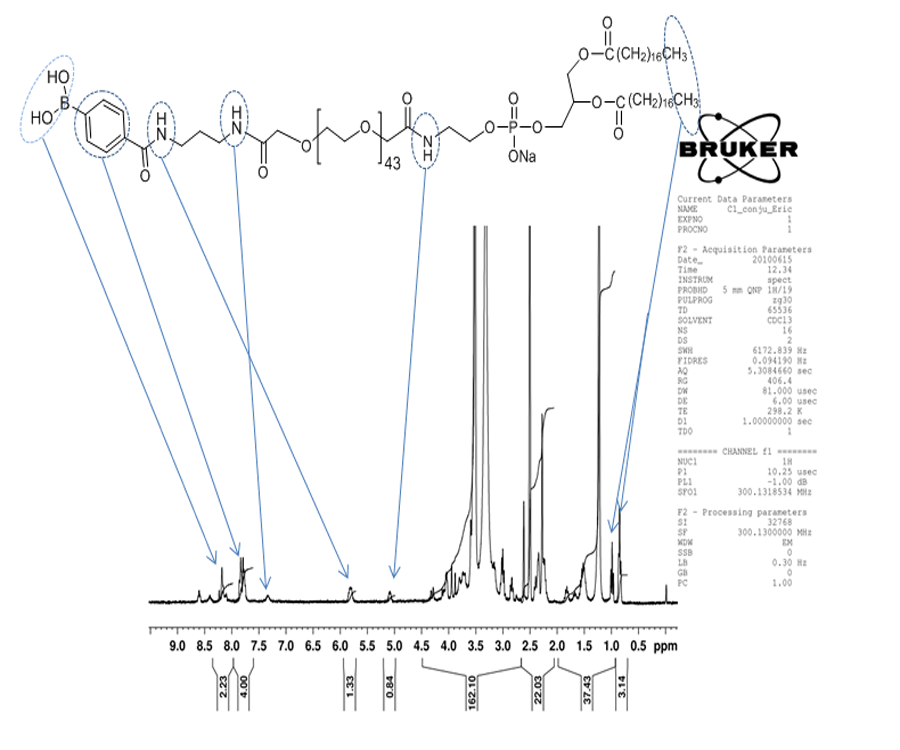

Supplement: Figure S3 — 4-aminocarbonylphenylboronic acid tethered to DSPE-PEG-COOH. (TIF) [file pone.0029585.s003.tif]
