# Supplementary material for: Non Inflammatory Boronate Based Glucose-Responsive Insulin Delivery Systems
Source: PLoS One. 2012 Jan 17;7(1):e29585. doi: 10.1371/journal.pone.0029585 (PMC3260138; doi:10.1371/journal.pone.0029585)
Supplement: Scheme S1 — General scheme for the preparation of PEGylated lipid-boronic acid conjugates. (DOC) [file pone.0029585.s004.doc]

**Scheme S1. General scheme for the preparation of PEGylated lipid-boronic acid conjugates.**
