# Supplementary material for: Non Inflammatory Boronate Based Glucose-Responsive Insulin Delivery Systems
Source: PLoS One. 2012 Jan 17;7(1):e29585. doi: 10.1371/journal.pone.0029585 (PMC3260138; doi:10.1371/journal.pone.0029585)
Supplement: Scheme S2 — General scheme for the preparation of PEGylated lipid-sugar conjugates. (DOC) [file pone.0029585.s005.doc]

**Scheme S2. General scheme for the preparation of PEGylated lipid-sugar conjugates.**
